# Resuscitating Patient Rights during the Pandemic: COVID-19 and the Risk of Resurgent Paternalism

**DOI:** 10.1017/S0963180120000535

**Published:** 2020-06-24

**Authors:** JOSEPH J. FINS

**Keywords:** COVID-19, resuscitation, patient rights, DNR, medical ethics, paternalism, history of medicine

## Abstract

The COVID-19 Pandemic a stress test for clinical medicine and medical ethics, with a confluence over questions of the proportionality of resuscitation. Drawing upon his experience as a clinical ethicist during the surge in New York City during the Spring of 2020, the author considers how attitudes regarding resuscitation have evolved since the inception of do-not-resuscitate (DNR) orders decades ago. Sharing a personal narrative about a DNR quandry he encountered as a medical intern, the author considers the balance of patient rights versus clinical discretion, warning about the risk of resurgent physician paternalism dressed up in the guise of a public health crisis.

## Plaintive Pleas

The doctors in the Emergency Department and across the hospital were imploring us to sanction unilateral do-not-resuscitate (DNR) orders, something that is not permitted under New York State law. However, in the throes of the pandemic, as we struggled to deal with a deluge of patients amidst scarcity, what they were asking made all the sense in the world.

By late March 2020, New York was in crisis mode. Our Emergency Department—like its counterparts across the City—was bulging at its seams. We were overwhelmed by the COVID-19 surge. Patients were arriving in respiratory failure at the cusp of needing to be resuscitated and the sheer volume of critically ill patients appearing at the same time was as if there had been a major plane crash at LaGuardia Airport. The only difference was that this was a sustained disaster with a steady flow of dying patients arriving at the hospital.

In addition, this was a *stress test* for medical ethics, for distributive justice and the allocation of scarce resources. Simply put, there were more patients to be resuscitated than available personnel, much less equipment. Yes, there were shortages of ventilators at the outset of the crisis, but more critically, there were not enough trained personnel to meet the demand for emergent intubations. Even at our large academic medical center, where we redeployed anesthesiologists who worked in the operating rooms and did research in labs, we were stretched thin. An anesthesiologist told me that during an overnight shift, it was not unusual to intubate 10 patients. A colleague of his intubated 13 patients one night, not all in the Emergency Department, but across the hospital.^1^ And that was just one physician’s efforts. So, it was incredibly busy, indeed, we were approaching the hinterland of chaos. Some might say battlefield conditions. So, why not allow unilateral decisions to not resuscitate patients who would inevitably die, even when families had refused? Why not allow practitioners who were stretched thin to provide care to those who might be saved?

There was also the pressing question of futility. By early April, we knew the mortality data from Wuhan for patients upon whom resuscitation was attempted. Only 2.9% survived a cardiac arrest.^2^ In a different sample from the United Kingdom, the Intensive Care National Audit & Research Center reported on 690 patients. As of April 3, 2020, the 30-day data mortality was equally distressing for patients who were intubated and in intensive care. Survival rates for all patients was 49.9% with mortality rates of 68.1% for patients 70 years and older.^3^ This is not ageism, but simply a report of the empirical data, begging the question of the futility or better yet, the *utility* of these interventions.^4^ Neither resuscitation nor intubation was a panacea. To think otherwise was to fall prey to what Joan Didion called *magical thinking.* ^5^

Of course, the complexity of resuscitation did not end with the narrow question of futility. There was the more nuanced proposition of proportionality, the relationship of risks to benefits, which can be described as a fraction, R/B. When the benefits are essentially zero, R/B can be expressed as the fraction R/0. If zero is in the denominator, by definition, the fraction is defined as infinite. When considering resuscitation, this would suggest that when there is no benefit of resuscitation, the risk is infinite and thus resuscitation constitutes unjustifiable risk.

One might counter that we cannot know for sure that resuscitation will be unsuccessful and therefore the degree of risk might be proportionate in some cases. Alternatively, one could argue that whatever risks might accrue have to be understood in a context where death is the alternative. These are fair arguments but miss a larger point: in a pandemic, proportionality is more complicated than a consideration of burdens and benefits as they devolve onto a patient.^6^ There are broader safety and resource allocation questions that must also be considered.

One of the major concerns regarding cardio-pulmonary resuscitation and intubation is the risk of aerosolization of the COVID-19 positive patient’s secretions. These can be highly infectious and all the more so when personal protective equipment is scarce or insufficient. So, when proportionality is considered in this broader context, although risks and benefits—such as they are—accrue to the patient, significant risks *also* accrue to clinicians performing the resuscitation. This is the time healthcare workers are most vulnerable to contagion.

As these issues were coming to the fore in New York, data from Spain about illness in healthcare workers were becoming available. Spain was ravaged by the corona virus with 18.5% contracting COVID-19.^7^ So, although the benefits of resuscitation for the patient exist in the realm of the hypothetical, the risk of illness for healthcare workers was quite real. Understood within a broader framework of benefits and burdens then, resuscitation becomes even more disproportionate because clinicians can become ill. This constitutes a personal harm to them and also has public health implications. If clinicians become sick in the performance of interventions without utility, they will be unable to provide care that is useful to other patients who might benefit from their ministrations.

All this would seem to make for an airtight case for unilateral DNR orders during a pandemic. I suspect they do and would not want the reader to think that I believe otherwise with crisis standards of care prevailing during a pandemic.

But, as I was taken by the logic of these arguments during the COVID-19 crisis, they made me uneasy. Something was amiss. Was this nod to physician discretion appropriate given the circumstances, or resurgent paternalism dressed up in the guise of a public health crisis? What about the hard-won choices of patients and families to make end-of-life decisions? Was this an erosion of those norms or simply a corrective to an autonomy ethic that had gone way too far? What Daniel Callahan described as self-determination run amok?^8^

I asked myself these questions and was comforted by what George Annas wrote decades ago critiquing the New York State DNR law as a national outlier. In a book on medical ethics, sponsored by the American Civil Liberties Union (ACLU),  Annas observed that, “It should be noted, however, that the State of New York has a law that mandates the use of CPR unless the patient or the patient’s proxy has refused it.^9^ This law improperly treats CPR as a unique treatment that patients will be subjected to without consent and seems to say that physicians must use it even when it is futile. But useless treatment is by definition no treatment at all and so should not be offered or performed…”^10^

It is hard to think that a book published by the ACLU, written by George Annas, and dedicated to Jay Katz^11^ could hardly be construed as illiberal or in any way an affront to patient’s rights. Annas has been a champion of this cause, and of course he is right about a legal mandate to provide care that would not work. But, it was a head and heart thing for me. Although I appreciated his stance and the coherence of the intellectual arguments I just espoused, I still felt a queasiness about advising colleagues about the permissibility of unilateral DNR orders.

At the simplest level, it was an aversion to giving the right advice in the wrong circumstance. At the outset of the pandemic, my colleagues were taking personal risks to serve our community, sometimes inadequately provisioned with personal protective equipment. How could I in good conscience advise them to go against the law? Was it right that they should risk their health and put themselves into legal jeopardy? Although Governor Andrew Cuomo did eventually introduce legislation into the state budget that provided some degree of legal immunity for medical practice under crisis standards of care^12^ ^,^^13^—another complicated aspect of the COVID-19 story about which I have published a full-length manuscript^14^—this modicum of protection only partly mitigated my concerns.

But my uneasiness was more than a question of legal vulnerability. It was something deeper and not just procedural concerns about adherence to the law. Although this was indeed important, and preoccupied my clinical colleagues, the source of *my* angst was different and much more personal. As the storm subsided over New York, I realized that my reservations were deeply linked to my personal narrative as a bioethicist. Indeed, the question of resuscitation during the pandemic brought me back to how I got into bioethics in the first place.

## Birth of a Bioethicist

It was over 30 years ago, but I remember the night vividly. I was asleep in the on-call room at Memorial Sloan-Kettering Cancer Center when a cardiac arrest was called. Interns generally do not go to codes. That responsibility is left to a code team of more experienced senior residents who are assigned the task of responding to and running resuscitation efforts. But this code was different. It was on my floor and, as such, I was supposed to respond. And, even more critically, it was one of *my* patients who was having a cardiac arrest.

So, when the nurse knocked on the doctor, I immediately went down to the patient's room. I can still see exactly where it was, at the corner of the building, overlooking York Avenue and the Rockefeller University across the street. Some moments are indelibly etched in one’s hippocampus and this was one of them. As I arrived at the room, one of the nurses told me that the patient’s attending physician had written a DNR order.

This seemed strange to me because earlier that afternoon, I had spoken to the patient about her wishes regarding end-of-life care and about resuscitation. She had wanted everything to be done, notwithstanding that she was in the final phases of multiple myeloma. So the attending’s order struck me as rather odd and inappropriate, given the patient’s wishes.

What should I do? I knew what she told me only hours earlier but the attending physician had written a DNR order. Could I possibly go against the orders of the attending physician, Dr. Burton J. Lee, who was also chief of the Myeloma Service and no ordinary attending? In short order, Lee, a Yale friend of George Herbert Walker Bush, would leave Memorial and become the White House Physician in 1988.^15^

Dr. Lee no doubt appreciated the futility of on-going care and felt that he was justified to write a unilateral DNR order without telling the patient. He might have been right about the futility of resuscitation, but it seemed improper to make the decision without consulting the patient or her family.

As I stood at the bedside and the nurse asked me what we should do, I was struck by fear and a sense of being utterly unprepared for this sort of decision and how to ascertain my moral commitments. Do I follow what I felt was an invalid order or do what I intuitively thought was the right thing to do? I had not studied bioethics—indeed, I dropped out of a bioethics class while a student at Wesleyan—and it had not been part of the curriculum while I was a medical student at Cornell. Unprepared as I was to make such a consequential decision, I was left to my own devices and rely upon my moral intuition.

I decided to attempt resuscitation despite the attending’s order. The patient did not make it and I went back to my on-call room convinced that I would be called into the chairman’s office the next morning to explain my actions. I worried that my short career in medicine would over before it had even begun because I had gone against the attending’s order. All night, I worried about what would happen to me. It was terrifying. I had done what I thought was the right thing to do but had been insubordinate.

I also realized that I had neither the language nor the analytics to defend my actions. None of us did in those days. In those wee-hours of the morning, it dawned on me that physicians needed better training to analyze moral choices in clinical practice. Although I did not appreciate it then, I was on my way to becoming a bioethicist. But that was in the future.^16^ All I was worried about the rest of the night was getting fired. When the next morning arrived, nothing happened. Remarkably, no one called me into the chairman’s office. There was no sanction, indeed no mention of my actions, ever. Paradoxically, I wondered if the outcome might have been different if the code had been successful and the patient survived.

So spared, I decided to become a bioethicist. Having survived this resuscitation, it seemed to me that I had an obligation to understand the ethical propriety of my actions and share what I learned with others. It was a formative night in so many ways.

## Resuscitation Redux

In retrospect, I came to appreciate that the night’s question about resuscitation was reflective of broader changes taking place in American medicine and that the orientations that Dr. Lee and I had reflected a generational shift between the waning days of physician paternalism and the ascent of patient autonomy.^17^

This dynamic was also playing out in New York state law when Governor Mario Cuomo signed the New York State DNR law in 1987.^18^ The law basically outlawed discretionary decisions about resuscitation by physicians. It required patients or their surrogates to consent to DNR orders. Without that consent, physicians were obliged to perform resuscitation.

Although criticized since, and viewed as problematic during the pandemic, when it was passed, the DNR law was viewed as progressive. It was seen as an endorsement of patient rights and a corrective to liberties taken at a number of hospitals in New York State, which designated patients not to be resuscitated without consent. A grand jury investigation in 1983 of LaGuardia Hospital in Queens found that physicians placed “small purple dots” on the medical charts of patients who they decided would not be resuscitated. Similar actions were taking place at Memorial Sloan–Kettering Cancer Center where I had done part of my internship. According to the *New York Times*, the New York State Department of Health investigators found that physicians there, “… wrote such ‘do-not-resuscitate’ orders in chalk on a blackboard, erasing them after they had been carried out.”^19^

Against this backdrop, Governor Mario Cuomo established The New York State Task Force on the Law to address questions at the interface of medicine, the law, and bioethics.^20^ In 1986, the Task Force issued a report outlining a legislative remedy to prevent surreptitious or covert decisions about resuscitation enacted without proper consent. The report noted that evasions like the purple dot system and slow or show codes “…were all means of achieving the results of a DNR order without risking the legal liability associated with issuance of the order. Each of these practices violates the physician’s professional obligations to patients and their families.” And perhaps most importantly, “… the furtiveness of these measures and the failure to obtain consent makes illicit and unethical what might otherwise be a medically appropriate decision.”^21^

This was the driving impetus for the law. It was neither a mandate to provide resuscitation nor state-sanctioned vitalism. The DNR report fully embraced the right to refuse life-sustaining therapy, but required the consent of the patient or family to forgo resuscitation. Unfortunately, the origins of the DNR amidst the scandal of “furtive” paternalism lead to an overreaction in the law, undermining even a reasonable degree of physician discretion. This led to the challenges faced during the pandemic and for calls to expand physician discretion both in New York and more broadly.^22^

There is a lesson in these distortions: When policies are born amidst a scandal or during a public health crisis, excess is often the result. Understood against the current convulsions of the COVID-19 pandemic, it is equally important that we do not overreact when we seek to modify the law and practice. We need to be careful about what we wish for lest our “reforms” undermine the hard-won rights of patients and families to direct care at life’s end. The frustration of clinicians forced to provide what were undoubtedly futile resuscitations makes the current climate of care ripe for resurgent paternalism, perhaps under a less offensive name, once the pandemic has passed. We will not be immune to the effects of the stresses placed upon clinicians during this time and how it informs moral judgment going forward.

Whether this is identified as resurgent paternalism or not, this trend could pose a threat to patient rights. This is something that is particularly worrisome as I write given the broader sociological dynamic of the COVID-19 and discussions of structural racism in the United States (and American medicine) following the murder of George Floyd in Minneapolis.^23^ It is important to note that many of the families who challenge unilateral DNR orders are marginalized by race or low socio-economic status.

A paper from the Massachusetts General Hospital where unilateral DNR orders are legal studied surrogates who persisted in requesting resuscitation after the medical team decided it was not indicated. Their paper, “After DNR: Surrogates who persist in requesting cardiopulmonary resuscitation” revealed that those who “persisted” in challenging the medical power structure and sought to counter a DNR order were disproportionately people of color, those for whom English was a second language or foreign born.^24^ The authors seemed not to notice this subset and the potential for implicit bias. In a commentary for the *Hastings Center Bioethics Forum* I noted that, “… although they [the authors] noted no significant difference between those who contested versus those who accepted a DNR decision ‘once the policy was invoked,’ they failed to comment upon the striking observation that the 19 patients whose surrogates rejected a DNR were disproportionately nonwhite (42.1%), foreign born (47.4%), and spoke a primary language other than English (15.8%).”^25^ I suggested that these demographics might have led to a sense of vulnerability on the part of surrogates and perhaps an implicit bias by the clinical team. These risks may be heightened when pandemic visitor restrictions limit family access to hospitalized patients and advocacy on their behalf.^26^

The legacy of health disparities and structural racism in our society, and in medical practice reflective of that society, suggest that the perception of surrogate opposition to DNR orders might be reflective of deeply seated power imbalances, prompting a need for surrogate resistance and advocacy on behalf of loved ones. Although these requests for care may result in futile interventions, it is important to appreciate what prompts these dynamics. I fear that giving clinicians a unilateral option to withhold resuscitation could truncate needed conversation and undermine mediation efforts.

At this moment in our history, both in the United States and abroad, as we grapple with systemic racism and seek to give voice to those who have burdened under constraints that compromise their ability for self-advocacy we should think very carefully about the rights of patients. The extreme conditions of a pandemic could easily become the template for a new normal that we might come to regret.^27^ This is bigger than the narrow purview of clinical ethics and speaks to the broader issues of social justice with which we must now contend.

## Notes

1. Personal Communication Dr. Peter Goldstein. June 12, 2020. E-mails in possession of the author.

2.

Shao F , Xu S , Ma X , Xu Z , Lyu J , Ng M , et al In-hospital cardiac arrest outcomes among patients with COVID-19 pneumonia in Wuhan, China. Resuscitation 2020;151:18–23 3228311710.1016/j.resuscitation.2020.04.005PMC7151543.

3. ICNARC Report on COVID-19 in critical care. Intensive Care National Audit & Research Center. April 4, 2020. ICNARC COVID-19 report 2020-04-04.pdf.

4.

Callahan D. Necessity, futility, and the good society. Journal of the American Geriatrics Society 1994;42(8)10.1111/j.1532-5415.1994.tb06559.x8046196:866–7.

5.

Didion J. The Year of Magical Thinking. New York: Vintage; 2007 .

6.

Fins JJ , Miller FG . Proportionality, pandemics and medical ethics American Journal of Medicine 2020;Jul 9:S0002-9343(20)30555-6. doi: 10.1016/j.amjmed.2020.06.008. Online ahead of printPMC734746732653418.

7. Red Nacional de Vigilancia Epidemiológica. Informe num. 22. Situación de COVID-19 en España a 13 de abril de 2020; available at https://www.isciii.es/QueHacemos/Servicios/VigilanciaSaludPublicaRENAVE/EnfermedadesTransmisibles/Documents/INFORMES/Informes%20COVID-19/Informe%20nº%2022.%20Situación%20de%20COVID-19%20en%20España%20a%2013%20de%20abril%20de%202020.pdf (last accessed 14 Apr 2020).

8.

Callahan D. When self-determination runs amok. Hastings Center Report 1992;22(2)1587727:52–5.

9. 1987 N.Y. Laws 818; N.Y. Public Health Law art. 29(B).

10.

Annas GJ. The Rights of Patients: The Authoritative ACLU Guide to the Rights of Patients. 3rd ed New York: New York University Press; 2004, at 291 .

11.

Katz J. The Silent World of Doctor and Patient. New York: The Free Press; 1984 .

12. Art. 30-D, N.Y. Pub Health Law, Sec. 3080.

13.

Hanfling D , Altevogt BM , Viswanathan K , Gostin LO , eds. Crisis Standards of Care: A Systems Framework for Catastrophic Disaster Response. Washington, DC: The National Academies Press; 2012 24830057.

14. Fins JJ. Sunshine is the best disinfectant, especially during a pandemic. *Health Law Journal of the New York State Bar Association.* Published on-line ahead of publication. Available at https://nysba.org/sunshine-is-the-best-disinfectant-especially-during-a-pandemic/ (accessed 1 Jun 2020).

15. Roberts S. Dr. Burton J. Lee, 86, Reagan AIDS Panelist and Bush’s White House Physician, Dies. *The New York Times*; 2016 December 1; available at https://www.nytimes.com/2016/12/01/nyregion/burton-lee-dead-white-house-doctor.html?searchResultPosition=1 (accessed 19 Jun 2020).

16.

Fins JJ. My Time in medicine. Perspectives in Biology and Medicine 2017;60(1):19–32 2889044610.1353/pbm.2017.0016.

17.

Fins JJ. A Palliative Ethic of Care: Clinical Wisdom at Life’s End. Sudbury MA: Jones and Bartlett; 2005 .

18.

Baker R , Strosberg MA , Bynum J. Legislating Medical Ethics: A Study of the New York State Do-Not-Resuscitate Law. Boston, MA: Kluwer Academic Publishers; 1995 .

19. Sullivan R. New York State drafts right-to-die guidelines. *The New York Times*; 1987 April 13; available at https://www.nytimes.com/1987/08/13/nyregion/new-york-state-drafts-right-to-die-guidelines.html?searchResultPosition=1 (accessed 19 Jun 2020).

20.

Institute of Medicine Committee on the Social and Ethical Impacts on Developments in Biomedicine. Society’s Choices: Social and Ethical Decision Making in Biomedicine. Washington, DC: National Academies Press; 1995 25121260.

21.

New York State Task Force on Life and the Law. Do Not Resuscitate Orders: The Proposed Legislation and Report of the New York State Task Force on Life and the Law. 2nd ed Berlin, Germany: Springer; 1988, at 7 .

22. Kramer DB, Lo B, Dickert NW. CPR in the Covid-19 Era—An ethical framework. *The New England Journal of Medicine* 2020. Available at https://www.nejm.org/doi/full/10.1056/NEJMp2010758?query=TOC

23. Evans MK, Rosenbaum L, Malina D, Morrissey S, Rubin EJ. Diagnosing and treating systemic racism. *The New England Journal of Medicine.* 2020. doi: https://doi.org/10.1056/NEJMe2021693

24.

Robinson EM , Cadge W , Zollfrank A , Cremens MC , Courtwright AM . After the DNR: Surrogates who persist in requesting cardiopulmonary resuscitation. Hastings Center Report 2017;47(1)10.1002/hast.66428074587:10–9.

25. Fins JJ. Who “persists” in opposing DNR orders? Demographics matter. *Hastings Center Bioethics Forum*; 2017 May 18; available at https://www.thehastingscenter.org/persists-opposing-dnr-orders-demographics-matter/ (accessed 19 Jun 2020).

26. Smith M, Vermylen J, Hauser J. “You can see your loved one now.” Can visitor restrictions during COVID unduly influence end-of-life decisions? Hastings Center *Bioethics Forum*; 2020 June 18; available at https://www.thehastingscenter.org/you-can-see-your-loved-one-now-can-visitor-restrictions-in-the-covid-era-unduly-influence-end-of-life-decision-making/ (accessed 19 Jun 2020).

27. Fins JJ. Pandemics, protocols, and the plague of Athens: Insights from Thucydides. *The Hastings Center Report* 2020;50(3):50–53.

